# Associations of air pollution with all-cause dementia, Alzheimer’s disease, and vascular dementia: a prospective cohort study based on 437,932 participants from the UK biobank

**DOI:** 10.3389/fnins.2023.1216686

**Published:** 2023-08-04

**Authors:** Shiqi Yuan, Xiaxuan Huang, Luming Zhang, Yitong Ling, Shanyuan Tan, Min Peng, Anding Xu, Jun Lyu

**Affiliations:** ^1^Department of Neurology, The First Affiliated Hospital of Jinan University, Guangzhou, Guangdong, China; ^2^Department of Intensive Care Unit, The First Affiliated Hospital of Jinan University, Guangzhou, Guangdong, China; ^3^Department of Clinical Research, The First Affiliated Hospital of Jinan University, Guangzhou, Guangdong, China; ^4^Guangdong Provincial Key Laboratory of Traditional Chinese Medicine Informatization, Guangzhou, Guangdong, China

**Keywords:** air pollution, dementia, genetic susceptibility, UK Biobank, cohort study

## Abstract

**Objective:**

To prospectively assess whether air pollution, including PM_2.5_, PM_10_, and NOx, is associated with the risk of all-cause dementia, Alzheimer’s disease (AD), and vascular dementia, and to investigate the potential relationship between air pollution and genetic susceptibility in the development of AD.

**Methods and results:**

Our study included 437,932 participants from the UK Biobank with a median follow-up period of over 10 years. Using a Cox proportional hazards model, we found that participants exposed to PM_2.5_ levels of ≥10 μg/m^3^ had a higher risk of developing all-cause dementia (HR = 1.1; 95% CI: 1.05–1.28; *p* < 0.05) compared to the group exposed to PM_2.5_ levels of <10 μg/m^3^. However, there was no significant association between PM_10_ levels of ≥15 μg/m^3^ and the risk of all-cause dementia, AD, or vascular dementia when compared to the group exposed to PM_10_ levels of <15 μg/m^3^. On the other hand, participants exposed to NOx levels of ≥50 μg/m^3^ had a significantly higher risk of all-cause dementia (HR = 1.14; 95% CI: 1.02–1.26; *p* < 0.05) and AD (HR = 1.26; 95% CI: 1.08–1.48; *p* < 0.05) compared to the group exposed to NOx levels of <50 μg/m^3^. Furthermore, we examined the combined effect of air pollution (PM_2.5_, PM_10_, and NOx) and Alzheimer’s disease genetic risk score (AD-GRS) on the development of AD using a Cox proportional hazards model. Among participants with a high AD-GRS, those exposed to NOx levels of ≥50 μg/m^3^ had a significantly higher risk of AD compared to those in the group exposed to NOx levels of <50 μg/m^3^ (HR = 1.36; 95% CI: 1.03–1.18; *p* < 0.05). Regardless of air pollutant levels (PM_2.5_, PM_10_, or NOx), participants with a high AD-GRS had a significantly increased risk of developing AD. Similar results were obtained when assessing multiple variables using inverse probability of treatment weighting (IPTW).

**Conclusion:**

Our findings indicate that individuals living in areas with PM_2.5_ levels of ≥10 μg/m^3^ or NOx levels of ≥50 μg/m^3^ are at a higher risk of developing all-cause dementia. Moreover, individuals with a high AD-GRS demonstrated an increased risk of developing AD, particularly in the presence of NOx ≥ 50 μg/m^3^.

## 1. Introduction

Dementia is a chronic, progressive, and ultimately fatal neurological disease. Currently, approximately 55 million people worldwide are affected by dementia, a number projected to reach 139 million by 2050 (Shin, 2022). While Alzheimer’s disease (AD) accounts for 60–70% of cases, most dementia patients present with a combination of AD, vascular dementia, and Parkinson’s disease-related dementia at the time of death (Kapasi et al., 2017). In addition to established risk factors like cardiovascular disease and an unhealthy lifestyle, the role of environmental factors in dementia pathogenesis is gaining attention (Cantuaria et al., 2021).

Air pollution, a significant global issue, has been linked to an increased risk of dementia in aging populations (Peters et al., 2019). Inhaled air pollutants have the potential to trigger an immune response (Genc et al., 2012; Parra et al., 2022). Exploring gene–environment interactions that contribute to the development of AD can aid in the development of personalized early intervention strategies to significantly reduce global AD incidence (Lane et al., 2018). In order to enhance prevention and treatment efforts, it is crucial to comprehend the relationship between air pollution, genetic risks, and AD (Sierksma et al., 2020). Polygenic risk scoring (PRS) techniques, which score individual genetic risks based on multiple genetic markers identified through genome-wide association studies (GWASs), have shown tremendous potential in identifying individual risks of developing AD (Leonenko et al., 2019). Certain investigations have utilized 21 AD-associated single nucleotide polymorphisms (SNPs) to compute AD’s genetic risk score (Zhang et al., 2023). However, with the advancement of genomics, an increasing number of AD-associated SNP sites have been discovered. Undeniably, incorporating a larger number of AD-associated SNPs is essential for the accuracy of an individual’s genetic risk assessment. Moreover, most studies have grouped air pollution levels into quartiles to investigate the association between air pollution and dementia (Chen et al., 2022; Parra et al., 2022; Zhang et al., 2023), without considering the potential non-linear relationship between air pollution levels and AD risk. Additionally, it is challenging for regression analysis in observational studies to incorporate all confounding factors. Therefore, choosing an appropriate method to correct potential confounding bias, such as inverse probability weighting (IPTW), is crucial for further verifying the reliability of the results (Imlay et al., 2020). Therefore, further rigorous research is needed to investigate the potential interaction between genetic factors and air pollution in the development of AD.

The objective of our study was to prospectively investigate potential non-linear relationship between air pollution (including PM_2.5_, PM_10_, and NOx) and the risk of all-cause dementia, AD, and vascular dementia. Additionally, we aimed to assess the relationship between air pollution and genetic susceptibility in the development of AD. Our study also provided an evidence-based medical rationale for individualized prevention of AD in participants with different genetic risks for AD.

## 2. Methods

### 2.1. Study population

The data used for this analysis were obtained from the UK Biobank (UKB). This prospective population-based cohort study involved over 500,000 individuals from various regions of the UK (Sudlow et al., 2015; Wu et al., 2021). The UKB study received approval from the Northwest Multicenter Research Ethics Committee, and all participants provided written informed consent (Sudlow et al., 2015; Yang et al., 2020). Our research project has been granted access to the UKB data (Application ID: 76636).

**Figure 1:**
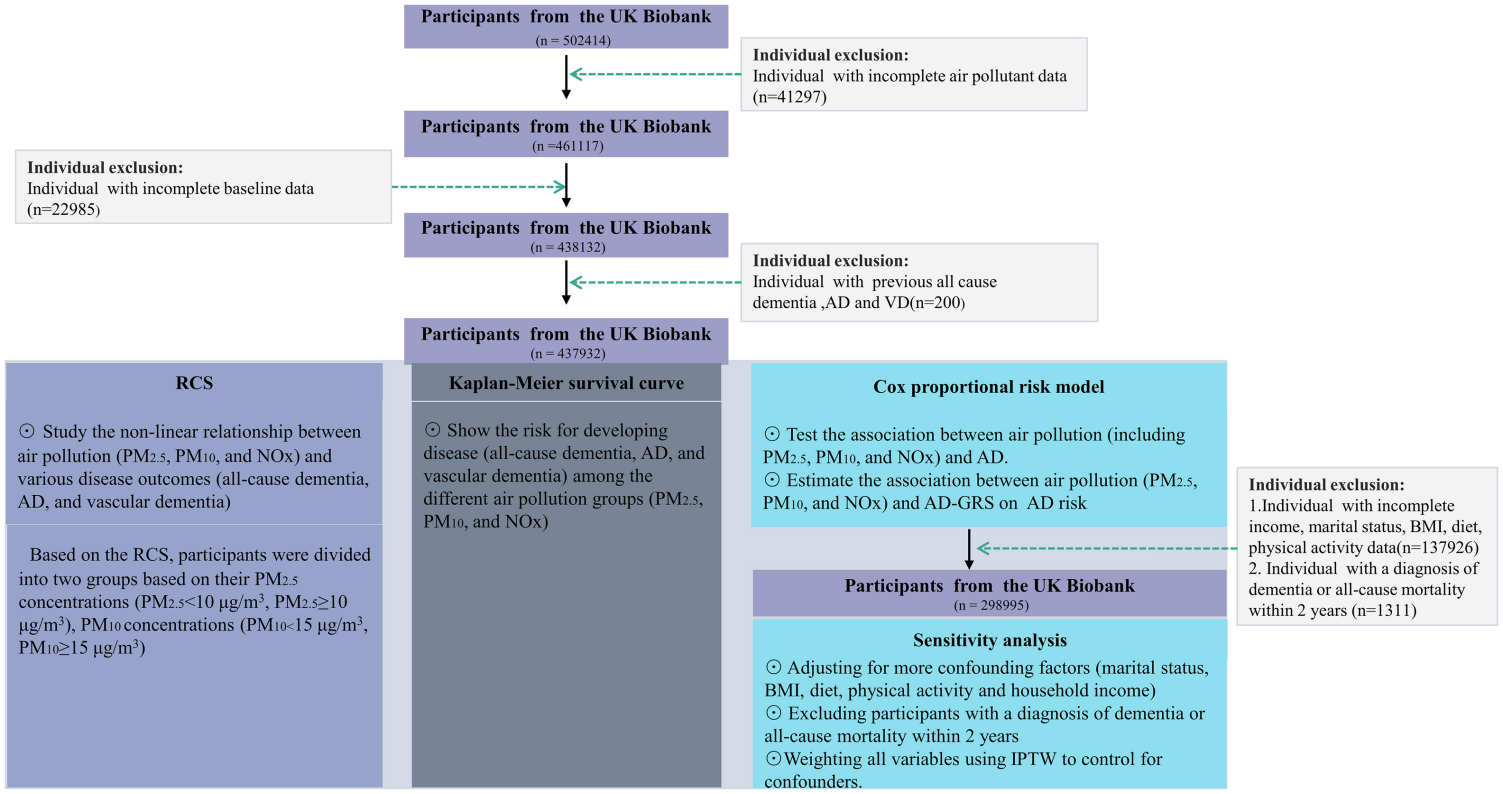
A flowchart of the study population. AD, Alzheimer’s disease; BMI, body mass index; RCS, Restricted cubic splines; NOx, nitrogen oxides; PM_2.5_, particulate matter with diameters ≤2.5 μm; PM_10_, particulate matter with diameters ≤10 μm. IPTW, Inverse Probability of Treatment Weighting.

Exclusion criteria for our study encompassed missing records of air pollutant data and other baseline information, as well as individuals with a pre-existing diagnosis of all-cause dementia, AD, or vascular dementia. A flowchart illustrating the study population is presented in Figure 1.

A total of 437,932 participants were included in our primary analysis, while our sensitivity analysis comprised 298,995 participants. All participants underwent a complete case analysis.

### 2.2. Assessment of exposure

PM_2.5_ and PM_10_ refer to particulate matter with diameters equal to or less than 2.5 and 10 μm, respectively. Measurements of air pollution levels for PM_2.5_, PM_10_, and NOx (nitrogen oxides) were obtained from the Small Area Health Statistics Unit,^1^ as part of the BioSHARE-EU Environmental Determinants of Health Project,^2^ in which the Biobank is a partner. The air pollution data were estimated based on the residential address of each participant at the scheduled follow-up date. The average concentration of air pollutants was determined using the 2010 European Study of Cohorts for Air Pollution Effects (ESCAPE) development and land use regression (LUR) model^3^ (Eeftens et al., 2012). The LUR model for air pollutants in 2010 was developed by ESCAPE monitoring conducted between January 26, 2010, and January 18, 2011, representing the year 2010 (Huang et al., 2021; Parra et al., 2022).

### 2.3. Outcome assessment

The determination of outcomes for all-cause dementia, AD, and vascular dementia in the UKB database is based on algorithmically-defined criteria.^4^ Information on death is obtained from the UKB’s death registration records. The most recent follow-up was conducted on March 23, 2021.

### 2.4. The definition of genetic risk score

In this study, to minimize false positives, newly discovered UKB loci were not included. We selected 29 independent SNPs that exhibited significant associations with AD from previous GWASs (Marioni et al., 2018; Jansen et al., 2019; Leng et al., 2021). The list of selected SNPs can be found in Supplementary material in Supplementary Table S1. For each individual included in the UKB, we determined an AD genetic risk score (AD-GRS) based on a previously published study (Fan et al., 2020). The effect size (b-coefficient) of each SNP was calculated using the reported GWAS results (Jansen et al., 2019). Each participant was assigned one of three categories of genetic risk for AD: high (quintile 5), intermediate (quartiles 2–4), or low (quintile 1).

### 2.5. Statistical analyses

Baseline characteristics were compared between the control groups (non-dementia, non-AD, non-vascular dementia, and survivors) and different outcomes (all-cause dementia, AD, and vascular dementia) using appropriate statistical tests. The Chi-square test was used for categorical variables, the Student’s t-test was used for continuous variables with a normal distribution, and the Wilcoxon rank sum test was used for continuous variables without a normal distribution. Continuous variables were reported as either the mean with standard deviation or the median with interquartile range (IQR).

To assess the non-linear relationship between air pollution (PM_2.5_, PM_10_, and NOx) and various disease outcomes (all-cause dementia, AD, and vascular dementia), a restricted cubic spline (RCS) model with 4 knots located at the 5th, 35th, 65th, and 95th percentiles was used. The number and position of the knots were determined based on Harrell’s suggestion for adequate model fit (Li et al., 2022). The RCS model was adjusted for age, Townsend deprivation index (TDI), sex, smoking, ethnicity, education level, and alcohol use. Participants were divided into two groups based on their concentrations of PM_2.5_ (PM_2.5_ < 10 μg/m^3^, PM_2.5_ ≥ 10 μg/m^3^), PM_10_ (PM_10_ < 15 μg/m^3^, PM_10_ ≥ 15 μg/m^3^), and NOx (NOx < 50 μg/m^3^, NOx ≥ 50 μg/m^3^) according to the RCS curve. Kaplan–Meier survival curves were used to visualize the risk of developing disease (all-cause dementia, AD, and vascular dementia) among different air pollution groups (PM_2.5_, PM_10_, and NOx). The log-rank test was employed to assess differences between the air pollution groups.

A Cox proportional hazards model was utilized to estimate the hazard ratios (HR) of PM_2.5_, PM_10_, and NOx in relation to different outcomes (all-cause dementia, AD, and vascular dementia). The proportional hazards assumption was tested using the Schoenfeld residual method, further details of the process can be found in Supplementary material in Supplementary Figure S1. Three models were constructed: Model 1 was unadjusted, Model 2 was adjusted for age, TDI, sex, smoking, ethnicity, education level, and alcohol use. In addition, the IPTW technique harmonizes the distribution of propensity scores across various cohorts, neutralizing the influences of confounding variables (Neumann and Billionnet, 2016; Wen et al., 2022). To minimize the influence of confounding factors on the outcomes, we employed the COX model after IPTW to verify the association between air pollution and AD. The IPTW variables included age, TDI, sex, smoking, ethnicity, education level, and alcohol use.

A Cox proportional hazards model was employed to investigate the association between air pollution (PM_2.5_, PM_10_, and NOx) and AD-GRS with respect to AD risk. The main analysis involved the following steps: Step 1: Participants were categorized into nine groups based on different combinations of AD-GRS (low, intermediate, and high) and air pollution levels (PM_2.5_, PM_10_, and NOx). We selected the low-level air pollution combined with low AD-GRS as the reference group to examine the association of the other groups with AD risk. Step 2: If the results from Step 1 indicated a significant difference in AD risk between low-level and high-level air pollution within a specific AD-GRS subgroup, we designated the low-level air pollution in conjunction with the corresponding AD-GRS as the new reference group to assess the association of the remaining groups with AD risk. The multivariable model was adjusted for age, TDI, sex, smoking, ethnicity, education level, and alcohol use. Additionally, several sensitivity analyses were conducted: (1) adjusting for additional confounders such as marital separation/divorce status (yes/no), body mass index (BMI, kg/m^3^), healthy diet (yes/no), physical activity (low/moderate/high), and average total household income before tax (categorized as less than £18,000/£18,000 to £30,999/£31,000 to £51,999/£52,000 to £100,000/greater than £100,000); (2) excluding participants with a diagnosis of dementia or all-cause mortality within a two-year period; (3) applying IPTW to all variables to control for confounders. The healthy diet score was based on factors such as beef/mutton intake, vegetable intake, fruit intake, fish intake, cereal intake, and sodium concentration in urine. Assessment methods for a healthy diet have been widely utilized in previous studies (Ma et al., 2019). A Cox proportional was used to estimate the hazard ratios for AD after IPTW, and the IPTW variables included age, BMI, TDI, sex, smoking, ethnicity, education level, marital separation/divorce status, healthy diet, physical activity, household income, and alcohol use.

All statistical analyses were conducted using the R package (version 4.1.0). A value of p below 0.05 was considered statistically significant.

## 3. Results

### 3.1. The baseline characteristics of control groups (non-dementia, non-AD, non-vascular dementia, and survivors) and different outcomes (all-cause dementia, AD, and vascular dementia)

Our study included a total of 437,932 participants, with a median follow-up duration of 12.01 years. Table 1 presents the outcomes observed at the end of the follow-up period: 2088 participants developed all-cause dementia, 778 participants developed AD, 432 participants developed vascular dementia, and 29,132 participants passed away. There were statistically significant differences (*p* < 0.05) in baseline characteristics between the control group and the various outcome groups, including age, TDI, sex, smoking, ethnicity, education level, and alcohol use.

**Table 1:** Baseline characteristics comparison between control groups (non-dementia, non-AD, non-vascular dementia, and survivors) and different outcomes (all-cause dementia, AD, and vascular dementia).

Characteristics	Outcome 1			Outcome 2			Outcome 3		
	Non-dementia	All-cause dementia	*p*	Non-AD	AD	*p*	Non-vascular dementia	Vascular dementia	*p*
Age (Median, IQR)	58 (50, 63)	65 (62, 68)	<0.001	58 (50, 63)	65.5 (62, 68)	<0.001	58 (50, 63)	66 (63, 68)	<0.001
TDI (Median, IQR)	−2.2 (−3.7, 0.4)	−1.8 (−3.5, 1.4)	<0.001	−2.2 (−3.7, 0.4)	−1.9 (−3.6, 1.3)	<0.001	−2.2 (−3.7, 0.4)	−1.3 (−3.2, 2)	<0.001
Sex (n, %)			<0.001			<0.001			<0.001
Female	236,503 (54.3)	933 (44.7)		237,067 (54.2)	369 (47.4)		237,279 (54.2)	157 (36.3)	
Male	199,341 (45.7)	1,155 (55.3)		200,087 (45.8)	409 (52.6)		200,221 (45.8)	275 (63.7)	
Ethnicity (n, %)			<0.001			<0.001			<0.05
White people	397,376 (91.2)	1920 (92)		398,580 (91.2)	716 (92)		398,904 (91.2)	392 (90.7)	
Mixed people	15,583 (3.6)	101 (4.8)		15,645 (3.6)	39 (5)		15,659 (3.6)	25 (5.8)	
Other people	22,885 (5.3)	67 (3.2)		22,929 (5.2)	23 (3)		22,937 (5.2)	15 (3.5)	
Education (n, %)			<0.001			<0.001			<0.001
College/University	140,472 (32.2)	405 (19.4)		140,740 (32.2)	137 (17.6)		140,809 (32.2)	68 (15.7)	
Other	295,372 (67.8)	1,683 (80.6)		296,414 (67.8)	641 (82.4)		296,691 (67.8)	364 (84.3)	
Smoking (n, %)			<0.001			<0.001			<0.001
Never	238,769 (54.8)	946 (45.3)		239,349 (54.8)	366 (47)		239,545 (54.8)	170 (39.4)	
Previous	152,130 (34.9)	911 (43.6)		152,704 (34.9)	337 (43.3)		152,841 (34.9)	200 (46.3)	
Current	44,945 (10.3)	231 (11.1)		45,101 (10.3)	75 (9.6)		45,114 (10.3)	62 (14.4)	
Alcohol (n, %)			<0.001			<0.001			<0.001
Never	18,731 (4.3)	153 (7.3)		18,816 (4.3)	68 (8.7)		18,853 (4.3)	31 (7.2)	
Previous	15,352 (3.5)	168 (8)		15,460 (3.5)	60 (7.7)		15,476 (3.5)	44 (10.2)	
Current	401,761 (92.2)	1767 (84.6)		402,878 (92.2)	650 (83.5)		403,171 (92.2)	357 (82.6)	
NOx (Median, IQR)	42.1 (34.1,50.6)	43.4 (35.4,52)	<0.001	42.1 (34.1,50.6)	43.4 (35.2,52)	<0.001	42.1 (34.1,50.6)	43.5 (36.7,51.9)	<0.01
PM_10_(Median, IQR)	16 (15.2,17)	16.1 (15.3,17.1)	<0.05	16 (15.2,17)	16.1 (15.3,17.1)	0.135	16 (15.2,17)	16 (15.3,17.1)	0.589
PM_2.5_(Median, IQR)	9.9 (9.3,10.6)	10 (9.4,10.7)	<0.001	9.9 (9.3,10.6)	10 (9.4,10.7)	<0.001	9.9 (9.3,10.6)	10 (9.4,10.6)	<0.05

AD, Alzheimer’s disease; IQR, interquartile range; TDI, Townsend deprivation index; NOx, nitrogen oxides; PM_2.5_, particulate matter with diameters ≤ 2.5 μm; PM_10_, particulate matter with diameters ≤ 10 μm.

### 3.2. The non-linear relationship between air pollution and all-cause dementia, AD, and vascular dementia

**Figure 2:**
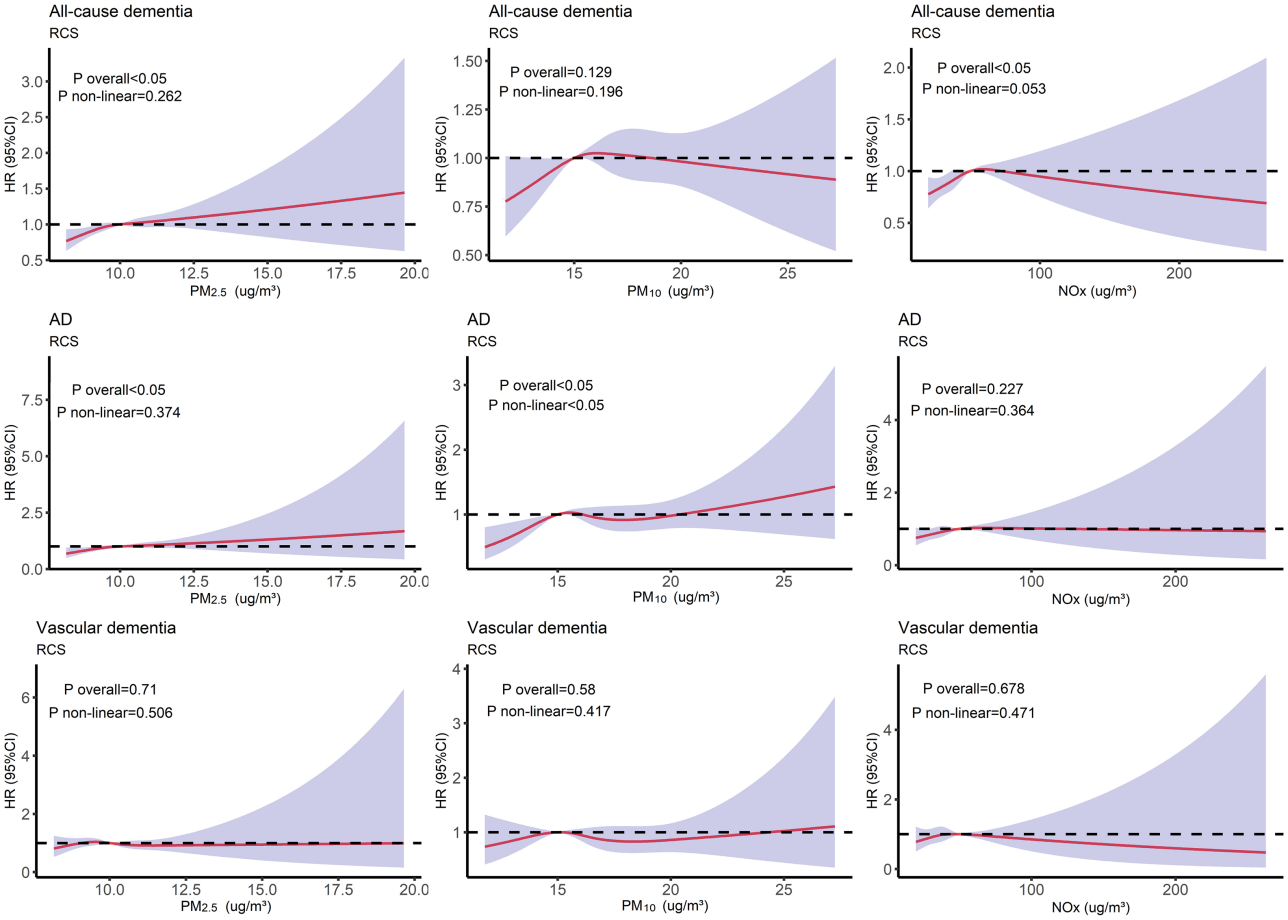
Restricted cubic splines (RCS) analysis depicting the non-linear relationship between air pollution (PM_2.5_, PM_10_, and NOx) and different outcomes (all-cause dementia, AD, and vascular dementia). Adjusted for age, TDI, sex, smoking, ethnicity, education level, and alcohol use.

**Figure 3:**
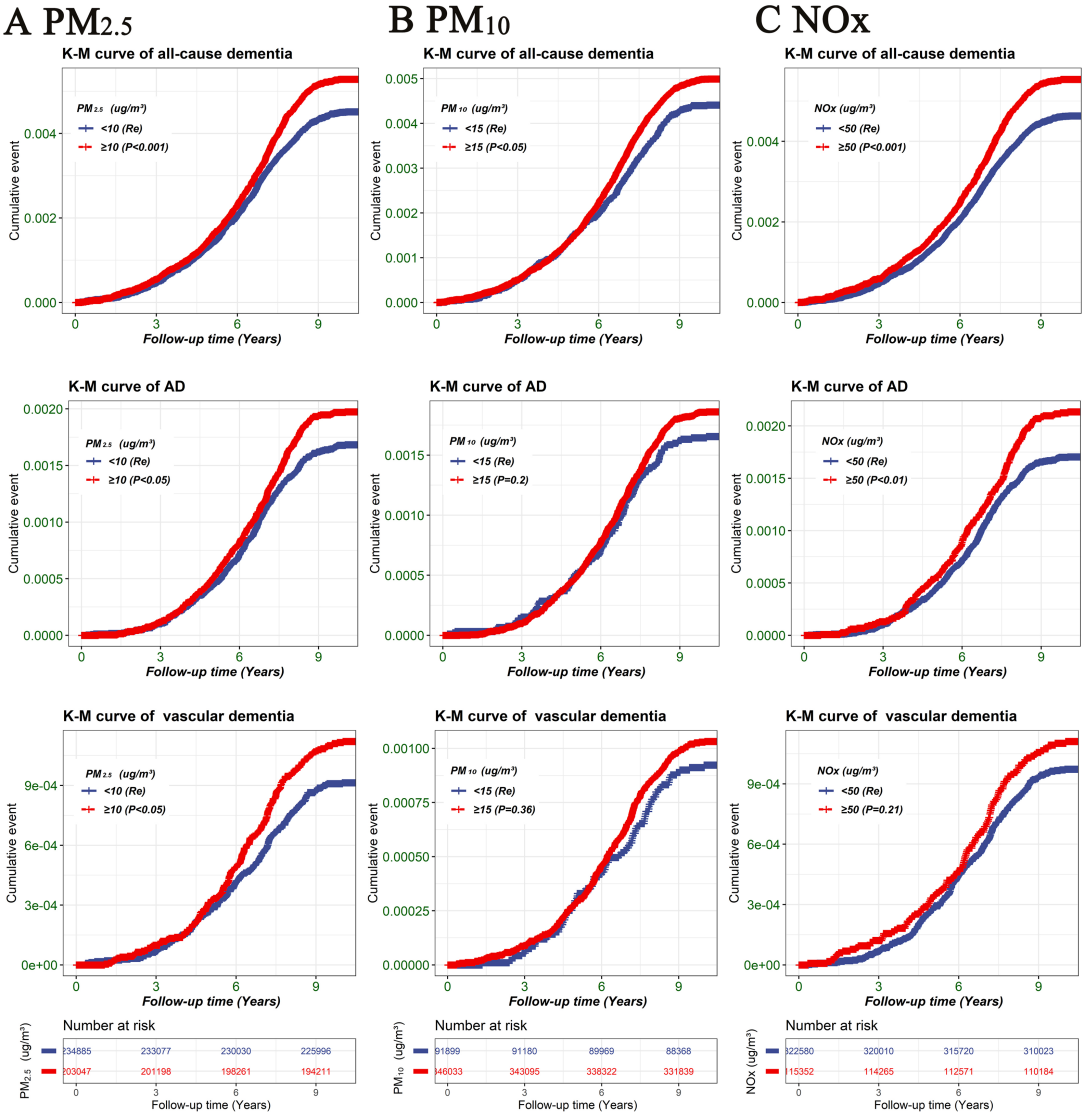
Kaplan–Meier survival curve illustrating the risk of different outcomes (all-cause dementia, AD, and vascular dementia) among air pollution groups (PM_2.5_, PM_10_, and NOx). **(A)** PM_2.5_, **(B)** PM_10_, **(C)** NOx. Differences between the air pollution groups were evaluated using log-rank tests. TDI, Townsend deprivation index; NOx, nitrogen oxides; PM_2.5_, particulate matter with diameters ≤2.5 μm; PM_10_, particulate matter with diameters ≤10 μm.

To investigate the non-linear relationship between air pollution (PM_2.5_, PM_10_, and NOx) and various outcomes (all-cause dementia, AD, and vascular dementia), an RCS analysis was conducted. Based on the RCS curve presented in Figure 2, participants were divided into two groups according to their concentrations of PM_2.5_ (PM_2.5_ < 10 μg/m^3^, PM_2.5_ ≥ 10 μg/m^3^), PM_10_ (PM_10_ < 15 μg/m^3^, PM_10_ ≥ 15 μg/m^3^), and NOx (NOx < 50 μg/m^3^, NOx ≥ 50 μg/m^3^). As illustrated in Figure 3, participants with PM_2.5_ concentrations of ≥10 μg/m^3^ exhibited higher risks of developing all-cause dementia, AD, and vascular dementia compared to those in the PM_2.5_ < 10 μg/m^3^ group. Additionally, participants in the PM_10_ ≥ 15 μg/m^3^ group had a higher risk of developing all-cause dementia compared to the PM_10_ < 15 μg/m^3^ group. Moreover, individuals exposed to NOx concentrations of ≥50 μg/m^3^ were associated with elevated risks of all-cause dementia and AD in comparison to those in the NOx < 50 μg/m^3^ group.

### 3.3. The hazard ratios for all-cause dementia, AD, and vascular dementia

A Cox proportional hazards model was utilized to estimate the HR for all-cause dementia, AD, and vascular dementia. As presented in Table 2, after adjusting for multiple variables (Model 2), the analysis revealed that participants with PM_2.5_ concentrations of ≥10 μg/m^3^ did not exhibit a significantly higher risk for AD or vascular dementia (*p* > 0.05). However, individuals in the PM_2.5_ ≥ 10 μg/m^3^ group were associated with a higher risk of developing all-cause dementia (HR = 1.1; 95% CI: 1.05–1.28, *p* < 0.05). In comparison to the PM_10_ < 15 μg/m^3^ group, there was no significant association observed between participants in the PM_10_ ≥ 15 μg/m^3^ group and a higher risk for all-cause dementia, AD, or vascular dementia. Conversely, participants exposed to NOx concentrations of ≥50 μg/m^3^ were significantly associated with an increased risk of all-cause dementia (HR = 1.14; 95% CI: 1.02–1.26, *p* < 0.05) and AD (HR = 1.26; 95% CI: 1.08–1.48, *p* < 0.05) compared to the NOx < 50 μg/m^3^ group. Similar results were obtained when conducting an analysis using IPTW with multiple variables (Supplementary material in Supplementary Table S2).

**Table 2:** Hazard ratios (95% confidence intervals) for all-cause dementia, AD, and vascular dementia.

	PM_2.5_ (μg/m^3^)			PM_10_ (μg/m^3^)			NOx (μg/m^3^)		
	<10(Re)	≥10	*p*	<15(Re)	≥15	*p*	<50(Re)	≥50	*p*
*All-cause dementia*									
Model 1	1.00(Re)	1.17 (1.00–1.28)	<0.001	1.00(Re)	1.13 (1.01–1.26)	<0.05	1.00(Re)	1.2 (1.09–1.32)	<0.001
Model 2	1.00(Re)	1.1 (1.05–1.2)	<0.05	1.00(Re)	1.05 (0.94–1.18)	0.366	1.00(Re)	1.14 (1.02–1.26)	<0.05
*AD*									
Model 1	1.00(Re)	1.17 (1.02–1.35)	<0.05	1.00(Re)	1.12 (0.94–1.34)	0.204	1.00(Re)	1.25 (1.08–1.46)	<0.01
Model 2	1.00(Re)	1.13 (0.97–1.31)	0.108	1.00(Re)	1.06 (0.88–1.27)	0.525	1.00(Re)	1.26 (1.08–1.48)	<0.01
*Vascular dementia*									
Model 1	1.00(Re)	1.23 (1.02–1.48)	<0.05	1.00(Re)	1.12 (0.88–1.42)	0.36	1.00(Re)	1.14 (0.93–1.41)	0.209
Model 2	1.00(Re)	1.04 (0.85–1.28)	0.689	1.00(Re)	0.98 (0.77–1.25)	0.879	1.00(Re)	0.94 (0.75–1.19)	0.625

Cox proportional hazard models were utilized to estimate the hazard ratios (HR) of PM_2.5_, PM_10_, and NOx in association with different outcomes (all-cause dementia, AD, and vascular dementia), using low-level air pollution as the reference group. Model 1 was unadjusted; model 2 was adjusted for age, TDI, sex, smoking, ethnicity, education level, and alcohol use. Re, reference; TDI, Townsend deprivation index; NOx, nitrogen oxides; PM_2.5_, particulate matter with diameters ≤ 2.5 μm; PM_10_, particulate matter with diameters ≤ 10 μm.

### 3.4. The joint association between air pollution (PM_2.5_, PM_10_, and NOx) and AD-GRS for developing AD

**Figure 4:**
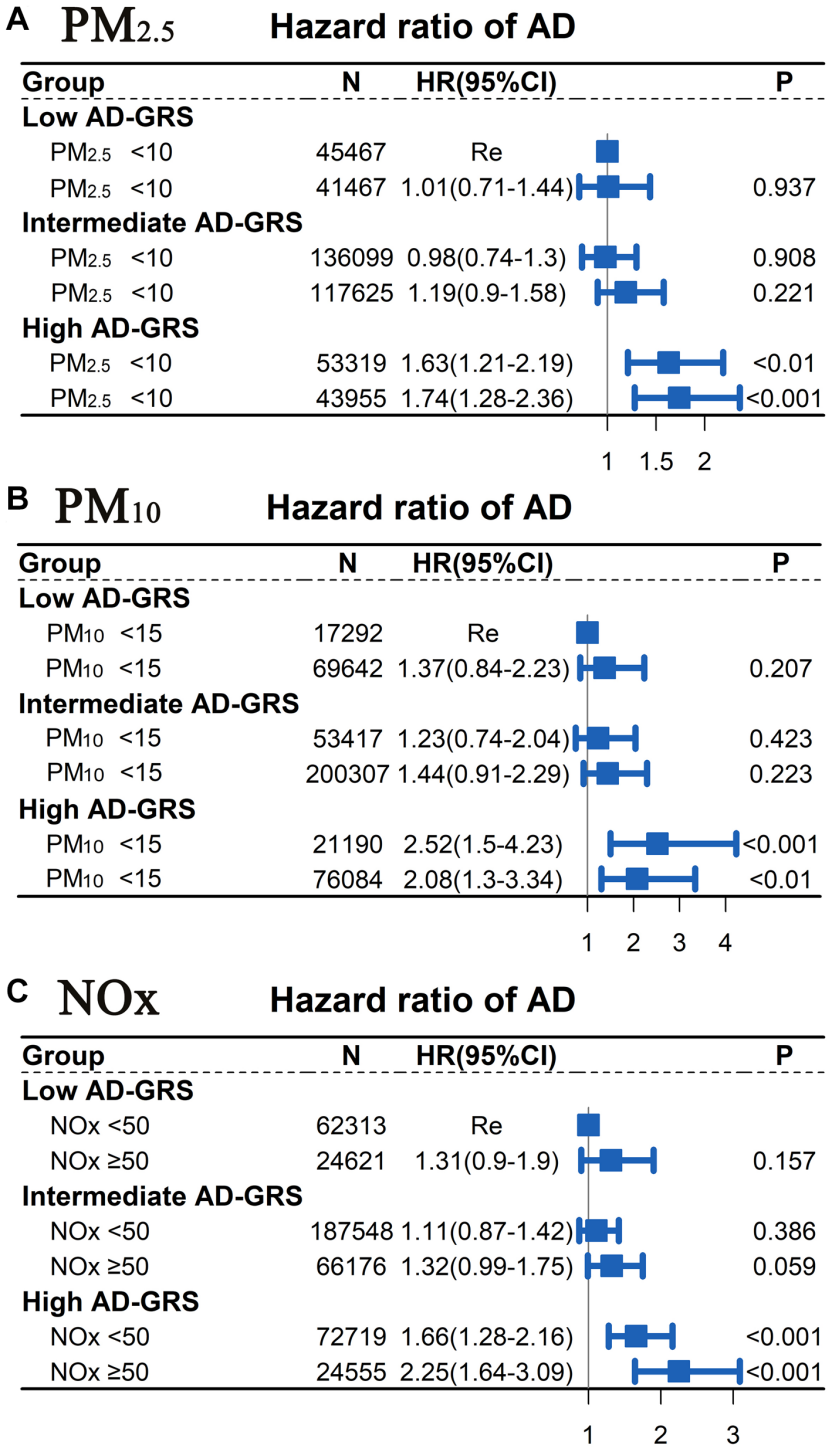
Joint association between air pollution (PM_2.5_, PM_10_, and NOx) and AD-GRS for AD risk assessed using Cox proportional hazard model. Low levels of air pollution [**(A)** PM_2.5_ < 10 μg/m^3^; **(B)** PM_10_ < 15 μg/m^3^; **(C)** NOx < 50 μg/m^3^] combined with low AD-GRS were chosen as the reference group. The multivariable model was adjusted for age, TDI, sex, smoking, ethnicity, education level, and alcohol use. TDI, Townsend deprivation index; NOx, nitrogen oxides; PM_2.5_, particulate matter with diameters ≤2.5 μm; PM_10_, particulate matter with diameters ≤10 μm.

A Cox proportional hazards model was employed to examine the joint association between air pollution (PM_2.5_, PM_10_, and NOx) and AD-GRS in relation to AD risk. The analysis did not reveal a significant interaction between air pollution and AD-GRS for AD risk (*p* > 0.05). As depicted in Figure 4, participants with PM_10_ concentrations of ≥15 μg/m^3^ or PM_2.5_ concentrations of ≥10 μg/m^3^ did not display a significantly higher risk of AD, regardless of their AD-GRS score, when compared to the PM_10_ < 15 μg/m^3^ or PM_2.5_ < 10 μg/m^3^ groups (*p* > 0.05). However, among participants with a high AD-GRS, those exposed to NOx concentrations of ≥50 μg/m^3^ exhibited a significantly elevated risk of AD in comparison to individuals in the NOx < 50 μg/m^3^ group (HR = 1.36; 95% CI: 1.03–1.18, *p* < 0.05; see Supplementary material in Supplementary Figure S2). Furthermore, participants with a high AD-GRS were significantly associated with a higher risk of AD regardless of the levels of air pollution (PM_2.5_, PM_10_, and NOx). Furthermore, the sensitivity analysis (Supplementary material in Supplementary Figure S3) showed similar results to Figure 4.

## 4. Discussion

After conducting an extensive population study based on a UKB follow-up spanning over 10 years, we have obtained noteworthy findings. First, participants belonging to the PM_2.5_ ≥ 10 μg/m^3^ group exhibited a heightened risk for all-cause dementia. Moreover, individuals in the NOx ≥ 50 μg/m^3^ group displayed a significantly elevated risk for both all-cause dementia and AD. Second, in the case of participants with a high AD-GRS, those within the NOx ≥ 50 μg/m^3^ group faced a significantly amplified risk of developing AD compared to their counterparts in the NOx < 50 μg/m^3^ group.

Mounting evidence suggests a direct correlation between increased exposure to air pollutants and the risk of dementia (Cacciottolo et al., 2017; Peters et al., 2019; Fu and Yung, 2020). In fact, in 2020, the Lancet Commission recognized air pollution as a modifiable risk factor for dementia (Livingston et al., 2020). PM_2.5_ arises from either natural source, such as wildfire smoke, pollen, and volcanic ash, or anthropogenic sources, predominantly stemming from fuel burning activities like thermal power generation, incineration, home heating, and vehicular emissions (Mortamais et al., 2021). The European Union established a limit target value for PM_2.5_ (Directive, 2008/50/EC) in 2015, setting it at 25 μg/m^3^, whereas the standard set by the US Environmental Protection Agency stands at 12 μg/m^3^. Our study employed RCS and Cox proportional hazard models, revealing a higher risk of all-cause dementia among participants in the PM_2.5_ ≥ 10 μg/m^3^ group. These findings hold potential as a reference for future dementia prevention strategies. NOx pollution encompasses nitric oxide (NO) and nitrogen dioxide (NO_2_) (Gomez-Garcia et al., 2005), both of which have detrimental effects on ecosystems and human health (Tian et al., 2013). Studies have demonstrated a clear association between NOx exposure and the incidence of dementia (Oudin et al., 2016). Similarly, our study indicates that individuals in the NOx ≥ 50 μg/m^3^ group face a heightened risk of all-cause dementia and AD. Consequently, reducing ambient air pollution plays a pivotal role in mitigating the disease burden associated with dementia (Andersson et al., 2018).

The etiology of AD remains unclear, but it is widely believed that both genetic and non-genetic factors play a role in its pathogenesis (Jiang et al., 2013). While genetic factors are inherent and cannot be modified, environmental contributors can be altered (Jiang et al., 2013; Ridge et al., 2013). Notably, the impact of environmental contributors on AD may vary depending on an individual’s genetic risk for the disease. Previous studies have primarily assessed the genetic risk of cognitive function by examining the presence or absence of the APOE 4 gene in populations (Calderon-Garciduenas et al., 2016; Martens et al., 2022; Calderon-Garciduenas et al., 2023). However, in our extensive population study, we adopted a novel approach by utilizing 29 independent SNPs significantly associated with AD from previous GWASs to evaluate the genetic risk of AD in the UKB population. We employed a polygenic genetic risk score for AD, allowing us to assess the genetic risk for AD and explore the interaction between air pollution and genetic risk on AD susceptibility. Among participants with a high AD-GRS, we observed a significantly elevated risk of developing AD among those exposed to higher levels of NOx (NOx ≥ 50 μg/m^3^). These findings suggest that individuals with a high AD-GRS should avoid exposure to NOx. It is plausible that air pollutants may directly reach the brain through the systemic circulation, gaining entry through the nasal pathway and crossing the blood–brain barrier (Genc et al., 2012; Mortamais et al., 2021). This process could potentially trigger microglial activation, induce inflammatory responses and vascular endothelial injury, and generate reactive oxygen species that inflict damage on the brain (Kioumourtzoglou et al., 2016; Hahad et al., 2020; Kriit et al., 2021).

Our study stands out as a long-term follow-up investigation conducted on a large population. Additionally, we recognize the strong correlation between genetic factors and the development of AD, and thus, we examined for the first time how this association intersects with exposure to air pollution. Finally, to minimize the influence of confounding factors on the outcomes, we employed the COX regression model and the COX model after IPTW to verify the association between air pollution and AD.

However, we must acknowledge the limitations inherent in our study. Firstly, it is important to note that our study was observational in nature, which prevents us from establishing a definitive causal relationship between air pollution and AD. There may exist confounding factors that we are unaware of, which were not accounted for in our model. Secondly, we relied on a single measure of air pollution at baseline, failing to consider potential fluctuations in air pollution levels prior to and following the enrollment of participants, as well as the possibility of participants migrating to different geographic areas. To partially address this limitation, we made the assumption that changes in air pollution indicators corresponding to geography occur over the long term and that most participants are likely to remain in their original geographic area or migrate to locations in close proximity. Additionally, our study utilized a large sample of data, which helps to mitigate some of these concerns. Nevertheless, future cohort studies should incorporate multiple measurements of air pollution taken at different time points to thoroughly investigate the effects of air pollution changes on participants’ cognitive functioning. Lastly, it is worth noting that the majority of our participants are of European descent. However, even though this study primarily focused on a specific population, it can still provide valuable insights as a reference when examining populations in other regions.

## 5. Conclusion

Participants residing in areas with PM_2.5_ ≥ 10 μg/m^3^ or NOx ≥ 50 μg/m^3^ exhibited a heightened risk of all-cause dementia. Moreover, individuals with a high AD-GRS demonstrated an increased risk of developing AD, particularly in the presence of NOx ≥ 50 μg/m^3^.

## Data availability statement

The original contributions presented in the study are included in the article/Supplementary material, further inquiries can be directed to the corresponding authors.

## Ethics statement

The studies involving human participants were reviewed and approved by Northwest Multicenter Research Ethics Committee. The patients/participants provided their written informed consent to participate in this study.

## Author contributions

AX and JL led the research design. SY, XH, and LZ reviewed the literature, mainly the analysis process and paper writing. YL, MP, and ST participated in the paper analysis. All authors participated in the revision of the paper and agreed to publish the paper.

## Funding

This study was funded by Guangdong Provincial Key Laboratory of Traditional Chinese Medicine Informatization (Grant no. 2021B1212040007).

## Conflict of interest

The authors declare that the research was conducted in the absence of any commercial or financial relationships that could be construed as a potential conflict of interest.

## Publisher’s note

All claims expressed in this article are solely those of the authors and do not necessarily represent those of their affiliated organizations, or those of the publisher, the editors and the reviewers. Any product that may be evaluated in this article, or claim that may be made by its manufacturer, is not guaranteed or endorsed by the publisher.

## Supplementary material

The Supplementary material for this article can be found online at: https://www.frontiersin.org/articles/10.3389/fnins.2023.1216686/full#supplementary-material

## Footnotes

^1^ http://www.sahsu.org/

^2^ http://www.bioshare.eu/

^3^ https://www.escapeproject.eu/

^4^ https://www.ukbiobank.ac.uk

## Abbreviations

AD, Alzheimer’s disease; AD-GRS, Alzheimer’s disease genetic risk score; GWASs, genome-wide association study; IQR, interquartile range; IPTW, Inverse Probability of Treatment Weighting; NOx, nitrogen oxides; PM_2.5_, fine particulate matter with diameter < 2.5 μm; PM_10_, particulate matter with diameter < 10 μm; RCS, restricted cubic spline; SNPs, single nucleotide polymorphisms; TDI, Townsend deprivation index; UKB, UK Biobank
