# Supplementary material for: Associations of air pollution with all-cause dementia, Alzheimer’s disease, and vascular dementia: a prospective cohort study based on 437,932 participants from the UK biobank
Source: Front Neurosci. 2023 Aug 4;17:1216686. doi: 10.3389/fnins.2023.1216686 (PMC10436530; doi:10.3389/fnins.2023.1216686)

**Table S1.** The single nucleotide polymorphisms (SNPs) that showed significant genome-wide association with AD.

| SNP | CHR | BP | A1 | A2 | P | BETA |
| --- | --- | --- | --- | --- | --- | --- |
| rs4575098 | 1 | 161155392 | A | G | 1.9E-10 | 0.016412 |
| rs6656401 | 1 | 207692049 | A | G | 2.58E-18 | 0.025014 |
| rs4663105 | 2 | 127891427 | C | A | 1.45E-44 | 0.031095 |
| rs10933431 | 2 | 233981912 | G | C | 7.62E-10 | -0.01544 |
| rs6448453 | 4 | 11026028 | A | G | 1.98E-09 | 0.014705 |
| rs7657553 | 4 | 11723235 | A | G | 0.044664 | 0.00484 |
| rs9269853 | 6 | 32550322 | A | C | 3.04E-08 | 0.013069 |
| rs9381563 | 6 | 47432637 | C | T | 1.99E-10 | 0.014451 |
| rs1859788 | 7 | 99971834 | A | G | 1.8E-15 | -0.0184 |
| rs11763230 | 7 | 143108841 | T | C | 5.37E-09 | -0.01577 |
| rs4236673 | 8 | 27464929 | A | G | 1.48E-19 | -0.02016 |
| rs11257242 | 10 | 11721119 | C | G | 8.11E-06 | -0.01001 |
| rs7935829 | 11 | 59942815 | G | A | 2.7E-15 | -0.0176 |
| rs10792832 | 11 | 85867875 | A | G | 4.5E-18 | -0.01948 |
| rs11218343 | 11 | 121435587 | C | T | 8.12E-12 | -0.03593 |
| rs12590654 | 14 | 92938855 | A | G | 1.32E-10 | -0.01483 |
| rs442495 | 15 | 59022615 | C | T | 1.22E-09 | -0.01372 |
| rs117618017 | 15 | 63569902 | T | C | 3.44E-08 | 0.018027 |
| rs59735493 | 16 | 31133100 | A | G | 3.73E-08 | -0.01299 |
| rs113260531 | 17 | 5138980 | A | G | 7.91E-10 | 0.019986 |
| rs28394864 | 17 | 47450775 | A | G | 1.68E-08 | 0.012302 |
| rs2632516 | 17 | 56409089 | C | G | 7.63E-07 | -0.01078 |
| rs8093731 | 18 | 29088958 | T | C | 0.026745 | -0.01774 |
| rs76726049 | 18 | 56189459 | C | T | 3.38E-08 | 0.056797 |
| rs4147929 | 19 | 1063443 | A | G | 4.43E-07 | 0.014413 |
| rs41289512 | 19 | 45351516 | G | C | 1.5E-278 | 0.206303 |
| rs76320948 | 19 | 46241841 | T | C | 4.08E-08 | 0.034933 |
| rs3865444 | 19 | 51727962 | A | C | 5.15E-09 | -0.01376 |
| rs6014724 | 20 | 54998544 | G | A | 5.38E-10 | -0.02289 |

**Figure S1.** The proportional risk hypothesis was tested by the Schoenfeld residual method for the COX proportional risk model constructed for PM_2.5_ (Figure A), PM_10_ (Figure B), and NOx (Figure C) in association with different outcomes (all-cause dementia, AD, and vascular dementia). Our results show that there are no significantly linear association between the duration of follow-up and the standardized Schoenfeld residuals (*p*>0.05), and therefore the air pollutants in this Cox model follow the proportional risk assumption. A: model 1 was unadjusted; model 2 was adjusted for age, TDI, sex, smoking, ethnicity, education level, and alcohol use; model 3 was the risk ratio of different outcomes through COX proportional risk model after IPTW. IPTW, Inverse Probability of Treatment Weighting.


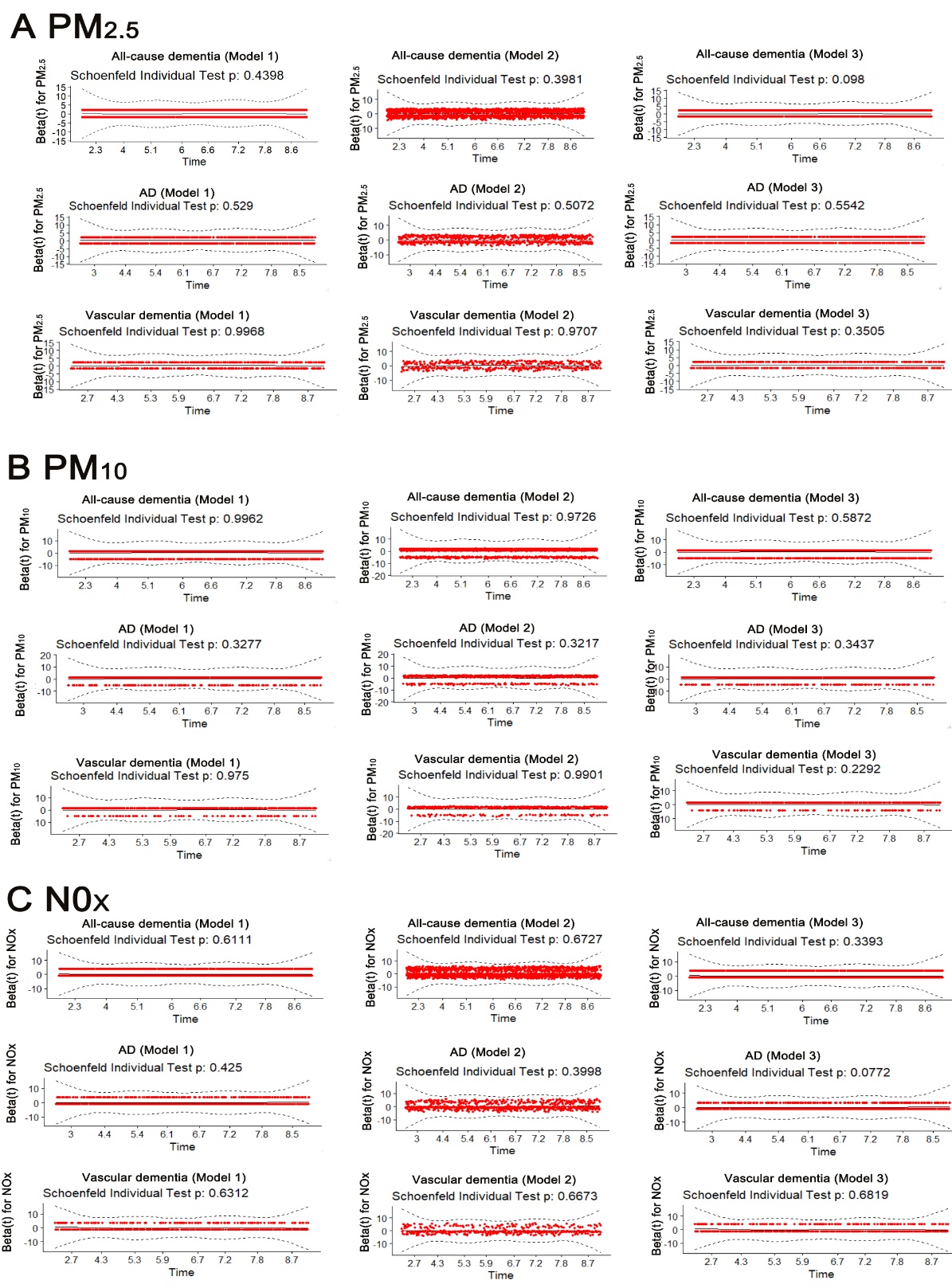


**Table S2.** Sensitivity analyses. The cox models represented the risk ratio of PM_2.5_, PM_10_, and NOx in association with different outcomes (all-cause dementia, AD, and vascular dementia) after IPTW, using low-level air pollution as the reference group. The IPTW variables included age, TDI, sex, smoking, ethnicity, education level, and alcohol use. Re, reference; IPTW, Inverse Probability of Treatment Weighting. TDI, Townsend deprivation index; NOx, nitrogen oxides; PM_2.5_, particulate matter with diameters ≤ 2.5 μm; PM_10_, particulate matter with diameters ≤ 10 μm.

|  | PM_2.5_ (μg/m^3^) | | | PM_10_ (μg/m^3^) | | | NOx (μg/m^3^) | | |
| --- | --- | --- | --- | --- | --- | --- | --- | --- | --- |
|  | **＜10(Re)** | **≥10** | ***P*** | ＜15(Re) | ≥15 | *P* | **＜50(Re)** | **≥50** | *P* |
| **All-cause dementia** | **1.00(Re)** | **1.09(1.02-1.19)** | **<0.05** | **1.00(Re)** | **1.05(0.75-1.17)** | **0.438** | **1.00(Re)** | **1.19(1.09-1.31)** | <0.01 |
| **AD** | **1.00(Re)** | **1.12(0.97-1.29)** | **0.15** | **1.00(Re)** | **1.07(0.9-1.28)** | **0.466** | **1.00(Re)** | **1.28(1.1-1.49)** | <0.01 |
| **Vascular dementia** | **1.00(Re)** | **1.03(0.85-1.24)** | **0.794** | **1.00(Re)** | **0.92(0.74-1.15)** | **0.543** | **1.00(Re)** | **0.99(0.8-1.23)** | 0.957 |

**Figure S2.** Joint association between NOx and AD-GRS for AD risk by Cox proportional risk model. Low levels of NOx< 50μg/m^3^ combined with high AD-GRS was chosen as the reference group. The multivariable model was adjusted for age, TDI, sex, smoking, ethnicity, education level, and alcohol use. Our result showed the NOx>50 combined with high AD-GRS group had a higher risk of AD compared to the NOx<50 combined with high AD-GRS group (HR= 1.36; 95% CI:1.03-1.18, *p*<0.05). AD-GRS, Alzheimer’s disease genetic risk score; TDI, Townsend deprivation index; NOx, nitrogen oxides; PM_2.5_, particulate matter with diameters ≤ 2.5 μm; PM_10_, particulate matter with diameters≤10μm;Re,reference.


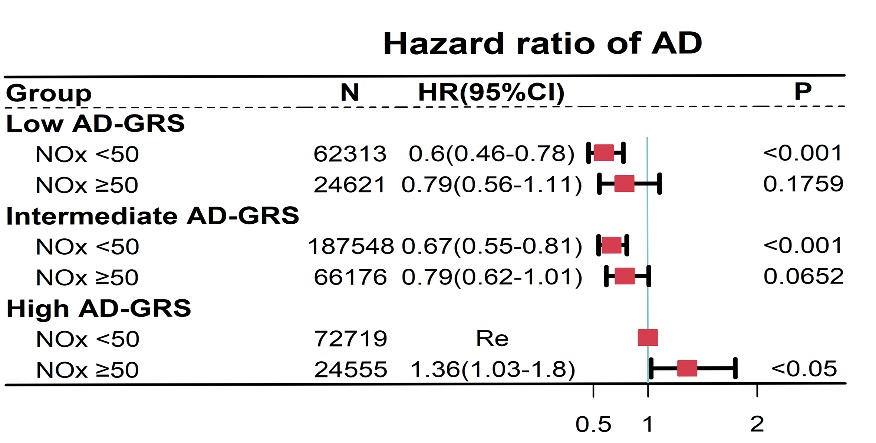


Figure S3. Sensitivity analyses. Several sensitivity analyses were conducted: 1) adjusting for additional confounders such as marital separation/divorce status, BMI, healthy diet, physical activity, and average total household income before tax; 2) excluding participants with a diagnosis of dementia or all-cause mortality within a two-year period; 3) applying IPTW to all variables to control for confounders. A Cox proportional was used to estimate the hazard ratios for AD after IPTW, and the IPTW variables included age, BMI, TDI, sex, smoking, ethnicity, education level, marital separation/divorce status, healthy diet, physical activity, household income, and alcohol use. Low levels of air pollution (A: PM_2.5_<10 μg/m^3^; B: PM_10_<15 μg/m^3^; C: NOx< 50 μg/m^3^) combined with low AD-GRS were chosen as the reference group. AD-GRS, Alzheimer’s disease genetic risk score; IPTW, Inverse Probability of Treatment Weighting. TDI, Townsend deprivation index; NOx, nitrogen oxides; PM_2.5_, particulate matter with diameters ≤ 2.5 μm; PM_10_, particulate matter with diameters ≤ 10 μm; Re, reference.


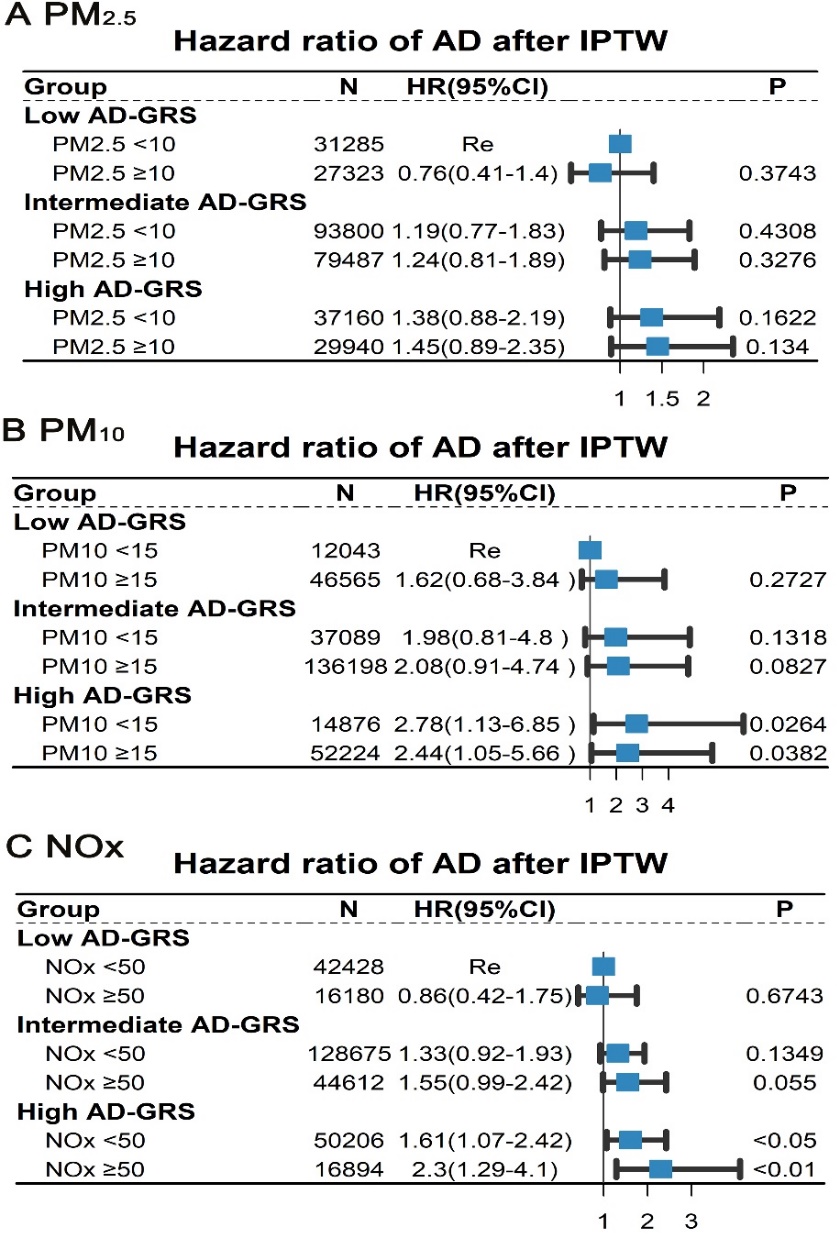

Supplement: Supplementary file 1 [file Data_Sheet_1.docx]
